# Understanding urban sustainability from Mode 2 Science and transdisciplinary education: how Master Thesis Ateliers of the Ghent *Stadsacademie* tackle wicked issues

**DOI:** 10.1007/s10668-022-02657-0

**Published:** 2022-09-09

**Authors:** Thomas Block, Charlotte Prové, Michiel Dehaene, Peter Vanden Abeele, Luce Beeckmans

**Affiliations:** 1 Stadsacademie, Centre for Sustainable Development, Department of Political Sciences, Ghent University, 9000 Gent, Belgium; 2 Stadsacademie, Department of Architecture and Urban Planning, Ghent University, 9000 Gent, Belgium; 3 Stadsacademie, City Government Architect, City of Ghent, Department of Architecture and Urban Planning, Ghent University, 9000 Gent, Belgium

**Keywords:** Urban sustainability, Wicked problems, Mode 2 Science, Transdisciplinary education, Sustainability education

## Abstract

The wicked sustainability problems that we are increasingly facing not only require new ways of knowledge production, but also challenge our traditional educational system. More and more importance is attached to educational practices and experiments focusing on transdisciplinary problem framing, a pluralistic search for solutions and active collaboration with various stakeholders throughout society. The aim of this article is to investigate how an inter- and transdisciplinary setting in which students develop master theses can contribute to learning about a specific urban problem and what challenges this transdisciplinary education entails. Starting from relevant theoretical and analytical frameworks, such as Mode 2 Science (Nowotny et al. 2005) and the three-phase model of Lang et al. (2012), we first outline the philosophy and approach of the general setting: the *Stadsacademie*, a collective learning platform or ‘collaboratory’ to explore and investigate wicked problems perceived in the city of Ghent (Belgium). To provide more in-depth and refined insights, we focus on an exemplary activity of the *Stadsacademie*: the Master Thesis Ateliers ‘Diversity in Social Housing’. A Master Thesis Atelier (MTA) is a collaborative trajectory of one academic year in which 4 to 8 master students and their supervisors from different disciplinary backgrounds concentrate on one specific urban problem and collaborate with non-academic actors aiming to explore and to impact upon that issue. We conclude this article with overall reflections and suggestions for transdisciplinary approaches within educational practices to tackle wicked sustainability issues.

## Introduction: focus on WREST problems

There is an emerging agreement that we are confronted with urgent and complex sustainability challenges (Latour, 2017; Raworth, 2017; Steffen et al., 2015). Problems such as climate change, loss of biodiversity, resource depletion, social inequality, poverty traps, etc. prove difficult to tackle, especially when there is (1) uncertainty regarding the knowledge base on which to frame and solve these complex problems; and (2) disagreement on norms and values. These kind of problems are often characterised and interpreted as so-called wicked problems (Rittel et al., 1973; Wiek et al., 2011; Dentoni & Bitzer, 2015; Hugé et al., 2016; Block et al., 2018). On the one hand, expert knowledge is often incomplete, fragmented, and uncertain, giving rise to scientific controversies, and on the other hand, social and political debate arises because of a lack of common ground on the world we want to live in and on the acceptability of goals and transition pathways.

Furthermore, sustainability issues have often fuzzy boundaries (while too often approached in monodisciplinary ways), are usually linked to each other and the many interactions between social and natural systems are of a high and increasing complexity (Nilsson et al., 2016; Ostrom, 2009). When such multi-faceted and multi-scalar problems link up with everydays needs, messy local contexts and genuine change, the concept of ‘real world problems’ is also used (O'Leary, 2005). When all of these characteristics are experienced let us refer to ‘Wicked, REal world SusTainability problems’ or abbreviated: WREST problems.

Acknowledging the specificity and wickedness of sustainability issues requires new ways of knowledge production (Kates et al., 2001). Since the mid-1990s, new modes of research have been increasingly promoted under labels such as ‘Mode 2 Science’, ‘Post-normal science’ and ‘Sustainability Science’ (Nowotny et al., 2005; Funtowicz & Ravetz, 1992; Komiyama & Takeuchi, 2006). One key aspect in these approaches is transdisciplinarity: the involvement of different scientific disciplines and non-academic actors into the research process in order to integrate and produce the best available knowledge and values, as well as create shared ownership to deal with WREST problems.

This wickedness also challenges traditional educational practices. The lack of an unambiguous scientific problem framing and clear-cut solutions is at odds with common conceptions of teaching and learning in terms of transferring reliable knowledge and acquiring specific skills and attitudes (Block et al., 2019; Sonetti et al., 2019; Van Poeck et al., 2019). At the same time, the idea is increasingly being raised to make education a vital driver for finding appropriate ways to deal with such WREST problems (Manring, 2014; UNESCO, 2017; Lönngren et al., 2016; Van Poeck et al., 2020). As such, increasing importance is attached to educational practices and experiments focusing on new ways of knowledge production and exchange, transdisciplinary problem framing, a pluralistic search for solutions and active collaboration with various stakeholders throughout society (Yarime et al., 2012; Lozano et al., 2013; Dentoni & Bitzer, 2015; Evans, 2015). Dealing with the diversity and controversy that characterise WREST problems also brings about ethical and political challenges within education (Östman, 2010; Van Poeck et al., 2019).

The aim of this article is to investigate into how a transdisciplinary setting in which students develop master theses can contribute to learning about a specific WREST problem and what challenges this transdisciplinary education entails. We try to address both research questions by providing an inspiring description and critical reflections on how Master Thesis Ateliers (MTAs) of the *Stadsacademie* try to tackle wicked urban sustainability issues.

The *Stadsacademie*^1^ (www.stadsacademie.be), founded in 2017 and embedded in Ghent University as an inter- and transdisciplinary consortium, functions as a collective learning platform or ‘collaboratory’ to explore and investigate WREST issues of the city of Ghent (Belgium). In doing so, the so-called academic brain power and the expertise of ‘the operational field’ are used together to address issues of the city in which the university is located, and thus offers an excellent opportunity to learn how to tackle WREST problems. Students, professors and researchers collaborate on specific issues with urban stakeholders among which policy makers, societal organisations, civil society organisations, citizen cooperatives, companies, artists, urban dwellers, etc. Although this transdisciplinary approach is part of a never ending endeavour and the educational practices of the *Stadsacademie* are being developed as we speak, this article wants to discuss the added value of the general philosophy of the *Stadsacademie* as well as of the MTAs, currently the cornerstones of the different trajectories within this collective learning platform.

A Master Thesis Atelier (MTA) is a collaborative trajectory of one academic year in which 4 to 8 master students and their supervisors from different disciplinary backgrounds concentrate on one specific urban WREST problem and collaborate with non-academic actors aiming to explore and to impact upon that issue. During this trajectory students not only receive intensive guidance and unique input from different actors, but they are also confronted with different perspectives and concerns as well as with their own blind spots through collective workshops, debates and field trips. Several MTAs have been running within the *Stadsacademie*, each with a specific focus: sustainable and urban food systems, urban diversity and social housing, circular economy, space for care and biodiversity. In order to provide more concrete and refined insights on MTAs, we focus in this article on the MTAs of one specific trajectory, namely the one on ‘Diversity in Social Housing’ that started in 2018 and already has three completed MTAs. At the moment, a fourth MTA is running.

In Sect. 2, we further explain ‘Mode 2 Science’ and transdisciplinary education as both form the basis of our analytical framework explained in Sect. 3 along with the methodology. Section 4 presents the philosophy of the *Stadsacademie* and the MTAs. In Sect. 5, we use the guiding question of our analytical framework to describe and critically reflect on the MTAs ‘Diversity in Social Housing'. In Sect. 6, we conclude the article with overall reflections and suggestions.

## Research and learning in transdisciplinary settings

The concept ‘Mode 2 Science’ allows us to comprehend that a transdisciplinary approach is an essential part of an alternative mode of knowledge production that is pre-eminently needed to tackle WREST problems. We connect this relevant concept with insights on transdisciplinary education, which we also discuss in this section to develop an integrated analytical framework with guiding questions in Sect. 3.

### Mode 2 Science and transdisciplinarity

Since the late 1980s, the idea that science as we know it is not responding adequately to the ‘grand challenges of our times’ and in particular those connected to sustainability has gained increasing acceptance with scientists and policy-makers. Sustainability's wicked character, its long-term horizon and the context in which sustainability challenges are to be addressed, result in specific demands for science (Kates et al., 2001; Ravetz, 2018).

In the mid-1990s, Gibbons and Nowotny introduced the terms ‘Mode 1 and Mode 2 Science’. While Mode 1 Science corresponds to the old paradigm of scientific knowledge production, Mode 2 Science is not only perceived as a concept, inherently open to manipulation or exploitation by others, but also as a project, an example of the social (re)distribution of knowledge which it seeks to describe (Gibbons, 1999; Gibbons et al., 1994; Nowotny et al., 2005). According to the ‘Mode 2’ knowledge production, it is essential to (1) acknowledge and describe the specific context and situation in which scientific problems arise, methodologies are developed, outcomes are disseminated and uses are defined; (2) follow a transdisciplinary approach, a form of learning and problem-solving involving cooperation between academics and non-academic actors; (3) keep in mind the greater diversity and the openness of the sites in which knowledge is produced, not at least because of new information and communication technologies; (4) be highly (self-)reflexive. Neutral observers (Mode 1) are replaced by engaged researchers who acknowledge multiple and contextual perspectives. Mode 2 relates to “a dialogic process, an intense (and perhaps endless) 'conversation' between research actors and research subjects” (Nowotny et al., 2005, p. 187); and (5) replace discipline-based peer review systems by novel forms of quality control in which experts from many disciplines, users, brokers and disseminators are involved and multiple definitions of quality are applied (not only ‘scientific excellence’ but also economic, political, social or cultural impact). These five characteristics embody the idea of the coproduction of socially robust knowledge and is closely linked to the recognition of one’s own limitations and assumptions. The latter is usually concealed or not acknowledged within Mode 1 Science. Acknowledging the limitations of all knowledge-production systems and the socially embedded, situated character of the genesis of scientific knowledge requires a modest attitude towards facts claimed by experts (Block et al., 2019; Latour, 1986). In Table 1, we summarise the essence of Mode 1 and Mode 2 Science.

**Table 1:** Idealtypical distinction between Mode 1 and Mode 2 (partly based on Gibbons, 1999; Gibbons et al., 1994; Nowotny et al., 2005)

	Mode 1	Mode 2
Knowledge is perceived as…	universal academic driven certain predictive neutral	context-sensitive application oriented uncertain explorative a coproduction of facts and norms
Approach is perceived as …	monodisciplinary one-sided academic technocratic	inter- and transdisciplinary pluralistic (multiperspectivism) academic and societal participative and experiential
Researchers are perceived as…	independent, neutral observers little reflexive hierarchically controlled controlled by peers (for publications)	engaged agents of change (self-)reflexive horizontally controlled controlled by extended peers (also users)

Parallel to the distinction between Mode 1 and Mode 2 Science, Funtowicz and Ravetz (1992) developed the concept of ‘post-normal science’ (PNS) which has attracted different conceptualisations, applications, and implications, ranging from being a ‘cure-all’ for democratic deficit to key to achieving more sustainable futures (Turnpenny et al., 2011, p. 287). Funtowicz et al. argue that applied science and professional consultancy are inadequate for addressing cases or issues where ‘facts are uncertain, values in dispute, stakes high and decisions urgent’. In a more recent publication, Ravetz (2018) fosters new thinking about PNS and states that complex sustainability problems cannot be resolved within the paradigm in which they are conceived. Other researchers use more or less identical arguments, but put forward different concepts such as ‘Sustainability science’ (Kates et al., 2001; Komiyama & Takeuchi, 2006), ‘Transformative sustainability science’ (Ravetz, 2018) or ‘Quadruple and quintuple helix frameworks’ (Carayannis et al., 2012).

**Fig. 1:**
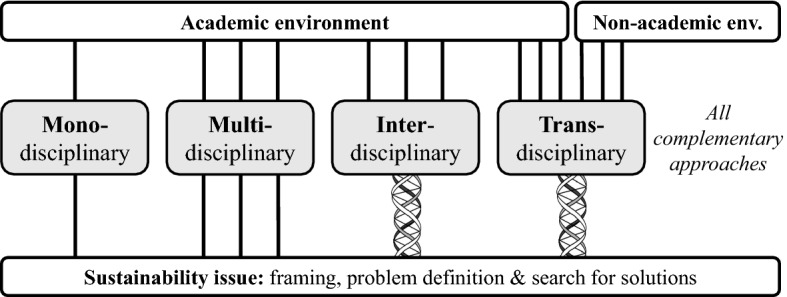
From monodisciplinarity to transdisciplinarity (Block et al., 2019, p. 33)

Despite differences in formulation and emphasis, all these approaches aim to acknowledge that sustainability-in-practice is the result of a coproduction process in which transdisciplinarity is crucial. For the sake of the focus of this paper, we will go into this latter characteristic more specifically. Transdisciplinarity is a reflexive, integrative, method‐driven scientific approach aiming to tackle WREST problems (and of related scientific problems) by differentiating and integrating knowledge that crosses disciplinary boundaries and involves cooperation between academics (including students) and other societal actors (Lang et al., 2012; Yarime et al., 2012; Dentoni & Bitzer, 2015; Block et al., 2019). As such, transdisciplinarity creates not only socially robust knowledge, but also legitimisation (Klein et al., 2001; Lang et al., 2012). As shown in Fig. 1, monodisciplinary research draws on only one scientific discipline to frame a problem and to search for solutions. A multi-disciplinary approach, in contrast, investigates an issue from the perspectives of several disciplines but without crossing the boundaries between these disciplines. The latter is different in interdisciplinary research characterised by cross-fertilisation between several disciplines. As mentioned, in transdisciplinary research also non-academic actors, expertise and experiences are involved in the production of knowledge around societally relevant problems.

We are committed to a common and comprehensive understanding of WREST problems and try to deal with different pools of knowledge or plural truth claims (cf. more theoretical approaches to transdisciplinarity), but our approach also aligns with problem solving approaches to transdisciplinarity (Klein, 2015; Renn, 2021). While a strict distinction between theoretical and problem solving approaches is not desirable, our premise is—as mentioned above—that WREST problems require new ways of knowledge production and that these kind of problems are best tackled through transdisciplinary settings.

### Transdisciplinary education on wicked issues

Teaching about so-called structured problems and clear answers concentrates on generally accepted and reliable knowledge. The main educational challenge then is to transfer this knowledge in a clear and understandable way. Since there are no clear-cut answers and single right solutions for WREST problems—often even not clear questions—teachers and educators are faced with other challenges.

As for WREST problems, many authors consider it as an opportunity for students to learn how to deal with a plurality of scientific positions, ontologies, framings, normative perspectives and ethical values. Students should have the skills to approach and handle sustainability controversies critically (Lundegård & Wickman 2012; Wiek et al., 2011; Evans, 2015; Östman et al., 2019). And also, the lack of clear-cut answers can at first sight seem problematic, but when interpreted as part of a ‘political’ struggle about the future of our societies, it opens up space for debate (Håkansson et al., 2019; Block et al., 2019). Furthermore, this pluralism should not lead to ‘anything-goes’ relativism or acquiescence, nor should it show understanding for alternative facts or fake news. Instead, it should allow to adopt a substantiated position as an engaged teacher, supervisor and/or student (Block et al., 2018). Teachers can reveal their own perspectives, concerns, doubts and value judgements. Doing this, they can stimulate students to explore the wicked issue, to confront the teacher’s perspectives with other perspectives, and finally, to form their own substantiated perspectives and concerns.

Ideally, this goes hand in hand with displaying a certain humility and modesty that not only exists in a less hierarchical role between teachers and students, but also relates to how to deal with scientific knowledge and to a willingness to learn from different actors. This implies to acknowledge that every scientific construction (in Mode 2 Science but also in Mode 1 Science) includes some concerns while excluding others (Jasanoff, 2004; Latour, 1986). So the framing of a sustainability issue by teachers also puts some elements at the forefront while leaving others out of view (Roberts & Östman, 1998; Evans, 2015; Block et al., 2018).

Discovering a plurality of perspectives, concerns, interests, values, abstraction levels, etc. works best when students are exposed to a concrete and/or local WREST problem (and related scientific questions). An abstract approach can provide theoretical frameworks and analytic concepts on transdisciplinary education, but it is only through challenge based learning on ‘real world problems’ or problem-oriented teaching that students are confronted with the wickedness of sustainability issues (Block et al., 2019; Manring, 2014; O'Leary, 2005; Östman et al., 2019; Walker et al., 2015). And this multiperspectivism is obviously best expressed when multiple students and their supervisors from different disciplinary backgrounds focus on a similar topic and interact in the same events. Such a group-based initiative has added value over a single student initiative in which one student works with a few supervisors and non-academic actors.

It is striking to notice that in comparison with transdisciplinary research, less attention is paid to transdisciplinary education (Balsiger, 2015). While inter- and transdisciplinary research is increasingly encouraged, education remains mainly organised in academic silos (Yarime et al., 2012; Wals, 2014; Deleye et al., 2019). As mentioned, solely academic knowledge is incomplete as a basis for addressing wicked issues. Tackling WREST problems in educational practices benefits from transdisciplinary education in which students are engaged in teaching and research that also integrates knowledge generated outside the academic world (Yarime et al., 2012; Lozano et al., 2013; Balsiger, 2015; Evans, 2015; Dentoni & Bitzer, 2015).

Despite its many advantages, inter- and transdisciplinary education is also facing some challenges and problems. Not only does it require theoretical, epistemological, methodological, and practical skills of both teachers and students to deal with multiple perspectives and methods and to dare to be modest and vulnerable, but developing transdisciplinary settings and interactions between academic and non-academic actors is also time-intensive and demands efforts in coordination, communication and logistics (Balsiger, 2015; Evans, 2015).

## Analytical framework and method

In this section, we use the central elements around ‘Mode 2 Science’ and transdisciplinary education as building blocks for the development of a framework with guiding questions to analyse our specific case. In the second part of Sect. 3 we explain our research method mainly based on case study research and focus groups.

### Towards guiding questions

In order to reflect and generate insights on transdisciplinary education practices, such as our MTAs, we interweave the central concepts, concerns and principles of Mode 2 Science and transdisciplinary education (see previous sections) with the three-phase model of Lang et al. (2012). The latter conceptualised a transdisciplinary research process as a sequence of three phases, but it is emphasised that in practice a cyclical and iterative process takes place. The guiding questions of Lang et al. (2012) serve as an excellent starting point because they focus on a transdisciplinary approach.

In our analytical framework, we modified these guiding questions by stressing the specific Mode 2 Science principles (see first column of Table 2): ‘context-sensitivity’ (i.e. acknowledge the specific context and situation of knowledge production), ‘application oriented’ (i.e. focus on a relevant ‘real world problem’), ‘uncertainty and explorative’ (i.e. deal with lack of clear-cut questions and answers of WREST problems), ‘pluralism and multiperspectivism’ (i.e. acknowledge a plurality of scientific positions, ontologies, framings, normative perspectives and ethical values), ‘engaged agents of change’ (i.e. deal with pluralism, but without falling into an ‘anything goes’ relativism or acquiescence), ‘reflexivity and modesty’ (i.e. acknowledge that every scientific construction around a wicked sustainability issue puts some elements at the forefront while leaving others out of view) and ‘broad quality control’ (i.e. change the peer review systems). And compared to Lang et al. (2012), our guiding questions strongly focus on educational practices and the role of students (see Table 2).

**Table 2:** Analytical framework

Mode 2 Science principles	Guiding questions
Applicable to the three phases:	*Phase 1: collaborative problem framing and building a research team*
Knowledge is perceived as: Context-sensitive application oriented uncertain and explorative a coproduction of facts and norms	1a. How to identify a relevant urban WREST problem together with all academic and non-academic actors involved, including the joint formulation of students’ research questions and their research design? 1b. How to build a transdisciplinary research team of engaged students, supervisors, other researchers and relevant non-academic actors that explores and tackles this WREST problem together?
Approach is perceived as:	*Phase 2: co-creation of solution-oriented and transferable knowledge through collaborative research*
Inter- and transdisciplinary pluralistic (multiperspectivism) academic and societal participative and experiential	2a. How to create relevant knowledge, a common focus and a shared understanding on the urban issue at stake? 2b. How to integrate different modes of knowledge production as well as the plurality of concerns, perspectives and values of different scientific disciplines and non-academic actors? What are the roles of all stakeholders involved? How to support students during each step of the collaborative research process?
Researchers are perceived as:	*Phase 3: re-integration of the insights in both scientific and societal practices*
Engaged agents of change (self-)reflexive horizontally controlled controlled by extended peers	3a. How to (re-)integrate the results in societal practices (e.g. policy recommendations, public debate, etc.) and in scientific practices (e.g. other research trajectories, publications, academic seminars)? 3b. How to integrate novel forms of quality control in which also non-academic actors are involved and not just purely scientific definitions of quality are applied?

We will use this analytical framework to describe and analyse the MTAs ‘Diversity in Social Housing’. The three phases and all guiding questions will be used to analyse our empirical material in Sect. 5. The Mode 2 Science principles will in turn structure our overarching reflections and conclusions in Sect. 6.

### Case study research and focus groups

In order to describe and reflect on the MTAs and to analyse the added value of learning in these transdisciplinary settings, we not only describe the general philosophy of the *Stadsacademie* and the MTAs, but we also focus on one specific trajectory with 3 MTAs. A single case can provide ‘rich data’ and as such a deep understanding of the exploring process (Flyvbjerg, 2006; Yin, 2014). As mentioned in the introduction, we base our study on the in-depth analysis of the MTAs ‘Diversity in Social Housing’. Not only are all authors of this article involved in these MTAs, but it is also a trajectory that has been ongoing for four consecutive academic years, which has enabled us to reflect on the process and to learn and adjust along the way.

Our empirical data comes largely from focus group sessions. Focus group research is a way of collecting qualitative data, which involves engaging a small number of people in an informal group discussion focusing on a particular topic (Onwuegbuzie et al., 2009; Wilkinson, 2004). More than with individual interviews, focus group sessions stimulate a dynamic discussion and is helpful to discuss perceptions, ideas, opinions, and thoughts. More specifically, our analysis is based on the sources of data mentioned in Table 3.

**Table 3:** Sources of empirical data

Source of data	Timing
Focus group session with students of this MTA in 2018–2019 and 2019–2020	February 22, 2021
Focus group session with students of this MTA in 2020–2021	February 15, 2021
Reflections and feedback of students during the MTA-trajectories	2018–2021
Focus group session with urban stakeholders of this MTA	March 3, 2021
6 discussion session on the philosophy of the *Stadsacademie* with academic partners and policy makers of the City of Ghent	December 2020–January 2021
Experiences from the authors as coordinator and driving force of this MTA	2018–2021

The students who followed and formed this MTA in the academic years 2018–2019, 2019–2020 and 2020–2021 were invited to participate separately in a focus group session (see Table 3). In yet another session, we also invited urban stakeholders such as the director of Ghent’s major housing association, the director of the Housing Department of the City of Ghent, the City Government Architect and the neighbourhood programme director. In total, 14 students and 6 urban stakeholders participated in these 3 focus groups.

Because of Covid-19, all sessions were organised online, lasted about 75–90 min and were moderated by the coordinator of the *Stadsacademie* (2nd author of this article), who can be considered more neutral because she is involved in the overall management of these MTAs and did not have to assess the students involved. We started with a briefing of the goal of the study and based ourselves largely on the guiding questions (see Table 2) to gain insight into the experiences and reflections of the students and urban stakeholders. We chose to analyse our data with a tape-based analysis, wherein the researchers listen carefully to the tapes of the audio-recorded focus groups and make transcripts on each relevant element of the analytical framework (Onwuegbuzie et al., 2009). All authors developed and discussed the coding scheme (largely based on our framework, see Table 2) and were involved in an intensive reading and re-reading of the transcripts. To increase the trustworthiness, the data of the focus groups were complemented with other sources of data, such as the experiences of all authors and several online discussion sessions with 32 academic and 16 non-academic partners focusing on the overall philosophy of the *Stadsacademie* (see Table 3).

## The *Stadsacademie* and the Master Thesis Ateliers ‘Diversity in Social Housing’

In this next section, we first elaborate on the philosophy and the organisation of the *Stadsacademie*. Then, we provide the necessary background to the specific MTAs ‘Diversity in Social Housing’ that we analyse in Sect. 5.

### The philosophy and organisation of the Stadsacademie

As mentioned in the introduction, the *Stadsacademie* focuses on WREST problems relevant to the city of Ghent. Through this ‘collaboratorium’, Ghent University engages to develop more structured cooperation with the city in which it is embedded. Academic actors (incl. students) and non-academic actors (but also ‘hybrid’ actors who have one foot in Ghent University and the other in the city administration) concentrate on (re)framing problems, defining new research questions, searching for innovative pathways, building future scenarios, developing experiments, etc. By grasping the synergy that is created between education, research and services to society, different relevant situations and problems are explored in-depth, multiple perspectives are taken into account, and an attempt is made to (re)integrate and apply the formulated socially engaged answers in both scientific practices at Ghent University and societal practices in the City of Ghent. Explaining the variety of trajectories and each concrete approach within the *Stadsacademie* is difficult within the contours of an article, which is why we concentrate on one exemplary example in the analysis (cf. Sect. 4.1).

The ambition of the *Stadsacademie* is to become an incubator and international reference for transdisciplinary research and education on urban WREST problems. The philosophy of the *Stadsacademie* is based on four principles that have already been explained in previous sections: acknowledging the wickedness; a focus on Ghent issues; a transdisciplinary process; and a synergy with education and student-led approaches.

The *Stadsacademie* was founded in 2017 by a small group of engaged professors and researchers who integrated the philosophy of the *Stadsacademie* into new and already running research and education practices. Among the activities are: *Stadsacademie*-sessions and MTAs, specific courses (or cases within a course) on WREST problems, research projects, summer schools, and valorisation activities such as exhibitions, public presentations, seminars, etc.

**Fig. 2:**
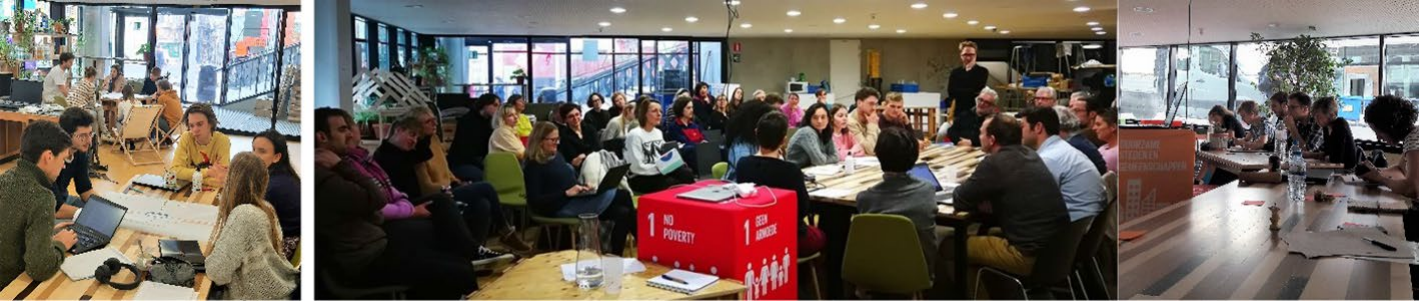
Pictures of the Stadsacademie space

Since 2020, the *Stadsacademie* is financially supported as an interdisciplinary consortium by the Research Department of Ghent University. This implies that a full-time coordinator has now been appointed. At the moment, the day-to-day management consists of this coordinator (2nd author) and three committed professors (including the 1st and 3rd author). The steering committee consists of several academic and non-academic actors (mainly Ghent policy makers, including the 4th and 5th author). The *Stadsacademie* also has a highly visible space in a university building in the city centre (see Fig. 2). Taken together, these recent changes ensure that (1) the *Stadsacademie* is not only a virtual organisation or a website but is spatially embedded in the context of Ghent, (2) that support and networks have broadened in the city (various departments) and in the university (across faculties), and (3) that the organisation of the *Stadsacademie* no longer only depends on voluntary engagements, but can now scale-up and broaden its scope thanks to the coordinator.

The *Stadsacademie* is structured around different trajectories, each of which focuses on a selected WREST problem. Currently, there are trajectories on social housing, urban food systems, mobility, circular economy, space for care and biodiversity.^2^ All research and education activities related to specific WREST problems are part of a trajectory. Other important aspects of the trajectories are: no predefined timeline, no expected output, and no fixed formats.

Generally, MTAs are the cornerstone of each trajectory. Networks are built in MTAs, knowledge is exchanged in inter- and transdisciplinary research teams, new insights and knowledge is generated, etc. A MTA usually starts with the organisation of a *Stadsacademie* session. These sessions are (semi-)public seminars or workshops where academics together with policy makers and other relevant urban actors from the city of Ghent try to identify key issues related to an urban WREST problem. The goal in this session is to formulate a shared ‘matters of concern’ (Latour, 2004) around which students, academics, and urban actors can collaborate in the MTA.

The MTA itself runs for 1 year, concentrates on a selected key issue and is a collaboration between 4 and 8 master students and their supervisors from different disciplinary backgrounds. In the MTA, non-academic actors are involved and contribute to the MTA to explore and impact upon the WREST problem. Each student focuses on one specific research question and methodology related to the key issue. Formally, the individually submitted thesis is always assessed according to the requirements of the specific master’s program of the student. At Ghent University, a master thesis counts for 20 to 30 ECTS credits (European Credit Transfer and accumulation System), with or without an integrated seminar or workshop track to stimulate the thesis progress (varies per master's program). This also means that the research question has to be in line with the educational program of the student, the evaluation of the thesis is done by the supervisor and the individual thesis has to fulfil the requirements of the faculty. Beside the specific research focus, students are given a (light) common assignment in which a problem framing is worked out and written out together around the issue at hand. In the MTA, multiple events are organised to stimulate exchange among the stakeholders: collective workshops, debates, lectures, field visits, etc. (see Sect. 5.2).

There are no formal procedures for organising a MTA. The purpose is that each MTA within a trajectory builds on the insights and the networks of the previous year. However, due to the refined or different focus on the WREST problem in the following academic year, a MTA has to look each year for supervisors, students and urban actors, all as volunteers.

### MTAs ‘Diversity in Social Housing’

In order to provide concrete and refined insights on MTAs, we focus in this article on the MTAs ‘Diversity in Social Housing’. The first MTA was organised in 2018 and currently the fourth edition is running. Table 4 provides specific information for the past academic years on the most important aspects of the MTA. We add more explanation and reflections in the next section of this article, but the overview in Table 4 already shows that a MTA needs time to grow in many areas.

**Table 4:** Overview of the MTA ‘Diversity in Social Housing’

Academic year	2018–2019	2019–2020	2020–2021
WREST problem	*Diversity in Social Housing*		
Specific focus	Development of an innovative housing program for refugees in Ghent	The social housing neighbourhood Watersportbaan	The social housing ‘garden city’ Sint-Bernadette
Coordination	Academics (2nd and 3rd author)	Academics (1st and 5th author) and ‘hybrid’ (4th author)	Academics (1st, 2nd and 5th author) and ‘hybrid’ (4th author)
Number of students	4	7	8
Involved educational programs	Masters of Science in Engineering-Architecture (all 4 students)	Masters of Science in Engineering-Architecture (3), Urbanism and Spatial Planning (2), Political Sciences (1), Geography (1)	Masters of Science in Engineering-Architecture (3), Urbanism and Spatial Planning (1), Political Sciences (2), Philosophy-Moral Sciences (1) Pedagogy-Social Work (1)
Involved actors (besides coordinators, students and their supervisors)	Academics from Ghent University and other universities, policy makers from Ghent (e.g. elderman and director of the housing department) and representatives from NGOs	Academics from Ghent University and other universities, policy makers from Ghent (e.g. from the housing department and the Policy Participation Unit) and representatives from social housing companies and NGOs	Academics from Ghent University and other universities, policy makers from Ghent (e.g. from the Urban Renewal Unit and the housing department) and representatives from social housing companies and NGOs
Events and activities (organised by coordinators)	One exhibition, one workshop, one lecture with debate	Two internal workshops, one site visit, one open workshop, one lecture with debate. The closing activity was cancelled due to COVID-19	Three internal workshops, one virtual site visit, one open workshop, one lecture with debate. All online due to COVID-19

The broad contours and potential challenges of the WREST problem of these MTAs were initially determined during a *Stadsacademie*-session in March 2018. Input on this session was given by academics from Ghent University and other universities, students, policymakers (e.g. eldermen), and representatives of NGO’s and housing cooperatives. In short, this problem is about the many challenges social housing in Ghent is facing today. These are mainly connected to two issues. On the one hand there is a severe shortage of social housing in Ghent, on the other hand, there is the social housing stock that requires an urgent renovation as it has been constructed in the interbellum period (e.g. neighbourhood Sint-Bernadette) or in the period after the Second World War (e.g. neighbourhood Watersportbaan). Moreover, the building stock is not only substandard to today’s energetic and constructive-technical norms, its design is also based on architectural and urban typologies that do not take into account today’s super-diverse demographic composition of Ghent. This situation further presses on the oversaturated private housing markets in the city, where especially low-income groups experience difficulties to find a proper home. In Flanders, there is a strong academic, societal and policy debate on the framing and transition paths around this complex challenge (De Decker et al., 2015). It is therefore desirable and necessary to expose in the MTA what is being put forward by whom, from what perspective and within what context. Table 5 (see Sect. 5.1) shows the various concrete research questions of academic year 2020–2021 that allowed to navigate agilely between different perspectives, to expose the perceived 'wickedness' and to explore collectively different pathways.

**Table 5:** Focus of students within MTA ‘Diversity in Social Housing’ in 2020–2021

Educational program	Research focus
Engineering-Architecture	Typological innovation in response to demographic variation in the Ghent neighbourhood Sint-Bernadette
Engineering-Architecture	Inclusive design scenarios for collective spaces in the Ghent neighbourhood Sint-Bernadette
Engineering-Architecture	Diversity-sensitive design for transitional spaces in the Ghent neighbourhood Sint-Bernadette
Urbanism and Spatial Planning	Alternative urban design strategies and instruments for supra-local connection and residential relocation from Sint-Bernadette
Political Sciences	Activism and urban policies: a comparative analysis focusing on patterns in two Ghent cases (‘Sint-Bernadette’ and ‘Craemersklooster’)
Conflict Studies	The impact of forced relocation on the citizenship of residents in the Ghent neighbourhood Sint-Bernadette
Philosophy-Moral Sciences	Social housing ethics: a moral deliberation on the forced relocation in the Ghent neighbourhood Sint-Bernadette
Pedagogy-Social Work	Urban sustainability and participation in social housing: an ethnographic case study research in the Ghent neighbourhood Sint-Bernadette

Although research within this MTA is always being conducted around this WREST problem, the focus is different every year, e.g. a specific Ghent social district or an urban challenge such as housing for vulnerable refugees (see Table 4). Also the coordination team of the MTA changes every year, yet the strength in this case is that the same coordinator has been taking the lead since 2018 (i.e. the 5th author of this article). This allows for continuity, stability in the networks and an ‘estafette’ effect (accumulation of insights, building further on previous year). The number of students and the number of educational programs involved was initially limited. In the first year, the four students were all involved in the same educational program. While the MTA strives for inter- and transdisciplinarity, the exchange between the students mainly concerned aspects from their own discipline. We have now reached a considerable number of students and an interesting mix of students, promoters, educational programs and other actors.

The activities mentioned in Table 4 only concern those organised by the MTA coordinators, but of course other urban actors also organised relevant debates, neighbourhood activities or protest actions. Students were often advised to attend these initiatives. In addition, students also organised numerous additional field visits and interviews with key figures to obtain relevant empirical data for their individual research.

## Analysis of the Master Thesis Ateliers ‘Diversity in Social Housing’

As mentioned, we will use our analytical framework with all relevant guiding questions (see Sect. 3) to describe and analyse the MTAs ‘Diversity in Social Housing’. We interweave feedback from students, coordinators, urban stakeholders with our own reflections. Overarching reflections and conclusions are discussed in the final section of this article.

### Collaborative problem framing and building a research team (Phase 1)

It is important to emphasise the preparation phase of the MTA. It usually takes a semester (or even a full year in the case of a new trajectory) to identify a relevant WREST problem and build a transdisciplinary research team. In the four years of the MTAs ‘Diversity in Social Housing’, we have learned that the selection of the specific annual focus and the building of the team happens simultaneously. It is an iterative and cyclical process in which the initial engagement usually starts from an academic actor who can assess the potential of a WREST problem because of familiarity with the topic, strong connections with the urban context and skills to organise and teach in a transdisciplinary setting. In this case, it is mainly thanks to the 5th author that we did not have to start from scratch and could build on existing expertise and Ghent networks. Nevertheless, it is each time a new quest for which no 3-step management plan is available. Reality shows an organic process in which numerous factors interact in a chaotic way, in our case: ongoing fundamental and action research, topics from existing educational practices, and last but not least, good contacts with certain urban key figures. “It's a small world. We just rolled into it” (quote urban stakeholder). Involved Ghent policy makers also indicate that the lack of strictly formal and politically supported processes to identify and select substantive themes on the one hand creates "a certain chaos" and "unclear roles in the beginning", but on the other hand it seems to be the only way to "structurally anchor necessary research trajectories around social housing […], a topic that is systematically understudied" (quotes urban stakeholders).

Yet, identifying a broad WREST problem does not mean that we already know the right specific questions for the MTAs. This is not a problem either, because our intention is precisely to work around issues that are not yet fixed, but open enough to allow for different perspectives and disciplines. As already mentioned, during the preparation phase of the first MTA, a *Stadsacademie*-session was organised in which academic and non-academic actors could put forward more refined concerns, issues or broad questions. In this way, the focus came on newcomers in the first year, which was broadened to vulnerable socio-economic groups in the next years. Due to Covid-19, we did not organise a session in the third year, and as such, the case ‘Sint Bernadette’ (cf. infra) was determined by the 4 coordinators of the MTA, but specifically at the request of the City Government Architect. Nevertheless, to experience the wickedness of the WREST problem and to involve an engaged network, it remains better to have the MTA preceded by or started with a broad participatory process that seeks a relevant and shared ‘matters of concern’ that has both academic and policy relevance.

We also learned that in order to consolidate and further develop the transdisciplinary research team, it is advisable to focus on an open, but also a concrete case. In our MTAs we therefore usually choose a neighbourhood, a campus or another type of urban space in or around Ghent. Such cases are tangible, they usually already receive policy attention, and they involve many different actors (citizens, activists, policy makers, residents, academics, civil society organisations, civil servants). These potential conflict spaces do not usually get the attention they deserve in the university or public debate because of their complexity and interdisciplinary nature. During the focus groups, many students indicated that a new world had opened up for them thanks to the MTA: “It pushed me with both feet deep into the practice of social housing and squatting, two worlds that were quite unknown to me” (quote student).

Our choice of open-ended topics and not predefined paths makes it challenging and difficult for the students: "In the beginning, the search process was tough", "It took me a long time to come up with a delineated research question" and "It was difficult to give own ideas a place in this MTA in the beginning" (quotes students). At the same time, students and other stakeholders also recognised the importance of serendipity. "It was fun and 'wise' to let the research question grow slowly by being there myself" and "I stumbled upon questions that I could not possibly have asked myself beforehand" (quotes students).

The MTA issues are undoubtedly attractive cases that students from different disciplines can address. Table 4 shows that in the beginning, mainly students from architecture and spatial planning were active in the MTAs, but gradually students from other programs have joined (e.g. political sciences, social work, moral philosophy, geography and conflict studies). We learned that it takes time for a MTA to grow and that precisely a more holistic approach is an important reason for joining an MTA: “Not only the subject, but also the opportunity to work beyond the walls of my own discipline was decisive for me” (quote student) and “Oddly enough, an MTA is a place where policymakers from different departments suddenly do get to meet” (quote urban stakeholder).

However, Ghent University still has a highly compartmentalised structure, in which faculties and programs often have their own emphases and administrative rules. This does not work in favour of an MTA. What complicates inter- and transdisciplinary cooperation within our MTA is the fact that master's theses in most faculties have to be developed individually and within one discipline. Not only does the final product then run counter to the overall intent of the MTA and the expectations of non-academic actors, but students are also not encouraged by this to engage in an inter- and transdisciplinary trajectory. To recruit students, we coordinators are currently tapping into our personal networks and promoting the MTA to younger students in our courses. Most students indicated during the focus group that they were aware of the MTA through students who had attended the MTA last year, through courses given by the MTA coordinators and through the *Stadsacademie* website. Hence the importance of holding a MTA several years in a row, so that students who have been made enthusiastic can take a MTA one or two years later. Yet every year there remains uncertainty about whether and which students will choose the MTA. At the moment, we are thinking about how to recruit more broadly in different educational programs through an appealing short video and putting up posters on all campuses.

Although a MTA is prepared a year in advance, the very specific concerns and research questions remains to be seen: which disciplines will be represented? What interests do the students (and their supervisors) have? How do these interests gradually (see Sect. 5.2) translate into research questions? It became clear that in the first 2 MTAs we focused too long on a broad and collective exploration for all students. Only after about three months did we leave enough room to formulate individual research questions from this common focus in line with the program the student is following. As a result, some students ran into problems to finalise their thesis in time. “Because of the collective focus in the beginning, I had clearly fallen behind other students who were not active in an MTA” (quote student). Since 2020, we ask students to formulate a preliminary research question after 1 month in consultation with their supervisor which can then, of course, be refined in a cyclical-iterative process.

Table 5 summarises the research focus (after 4 months) for each student in the MTA on the social housing ‘garden city’ Sint-Bernadette. In July 2020, it was decided to demolish this Ghent neighbourhood of 200 social housing units and to rebuild it afterwards. The following overview makes clear how forced relocations in and the rebuilding of a social housing neighbourhood can be approached from different disciplines.

A final element that is crucial for the first phase concerns clear agreements on roles and engagements within a MTA. You need at least some 'ambassadors', both from the university and from the city. In our case, we achieved a rather balanced structure between academic and non-academic partners thanks to the strong involvement of the 4th author, who is not only a visiting professor at Ghent University, but also the Ghent Government Architect. Together with the first and last author, he takes care of the content coordination of this MTA. This coordination implies not only the organisation of numerous activities (see Sect. 5.2), but also networking, communication and a permanent follow-up in order to keep the approach ‘common’. We have also learned that it is crucial to discuss and define the different roles and responsibilities of all other actors in advance. It cannot be the intention that some promoters send their students to the MTA without showing commitment to the collective element and in this sense just leave most of the work to the MTA coordinators. Not only is this uncollegial, but the involvement of all promoters from different disciplines is necessary to ensure a broad feedback to all students and to contribute to the joint process of inter- and transdisciplinary knowledge production.

### Co-creation of solution-oriented and transferable knowledge through collaborative research (Phase 2)

In order to strengthen the common understanding on the yearly topic and to interweave the available knowledge, the coordinators of these MTAs organise 5 to 7 collective moments each year with all the students, their supervisors and numerous other academic and non-academic actors. We try to develop a 'collaboratorium' by using different formats: a kick-off meeting in which the philosophy of the *Stadsacademie* and the MTA is outlined, in which the yearly topic is presented by urban stakeholders and the MTA coordinators, in which students get to know each other, etc.; a field visit during which the site is walked around and relevant explanations are given at various locations, e.g. by community workers, neighbourhood organisations, policy makers, academics, etc.; lectures and debates with key figures, often student-led (i.e. the students invite the speakers and give their questions in advance); speed dates where students can have a 1-on-1 short conversation with a wide range of urban stakeholders; workshops with all promoters where the students present and discuss their preliminary results; feedback sessions in which the work of each student is discussed individually by all promoters; etc. Due to COVID-19, we omitted the planned common assignment (see Sect. 4.1) in 2020–2021, but we believe that the inter- and transdisciplinarity of the MTA can be enhanced by having students collaborate and write a short joint paper (e.g. a policy recommendation, a problem framing, a manifesto or blog text). This is the only text they have to write together and in which they can actively experience that it is not easy to reconcile multiple perspectives, assumptions, views, normative preferences, etc. "It is challenging, but fascinating to work on a common assignment to which each student contributes from their own training" (quote student).

Our experience within the MTAs has already shown that a shared concern is not sufficient to guarantee collaboration and co-creation. For a constructive dynamic, it is crucial to learn about each other's struggles in dealing with different perspectives, each other's expectations in the process, and each other's working environment (e.g. how it functions, processes, limitations). Among others, a MTA can help to learn and acknowledge the typical logics of the academic and policy world. In the focus group sessions, one urban stakeholder expressed how she adjusted her expectations of academic actors once she understood better how universities operate. Also the students repeatedly stated that the MTA was not only an important source of information and opened the door to interesting contacts, but that it also gave them insight into policy perspectives around the WREST problem “As an architecture student, you are taken out of your tower, your ideas are actually tested by policy makers and other urban actors" (quote student).

And of course, students indicated that it is enriching to see how students and supervisors from other disciplines place different emphases in the research process: the importance of theoretical starting points differs, the use of analytical frameworks is not common everywhere, making one's own normative standpoints explicit is not equally stimulated everywhere, etc. And this also applies to the wide range of methods students chose to tackle the same issue within an MTA, for example in 2020–2021: a literature review; participant observations; urban design; architectural design; and moral case deliberation. At the same time, some students indicated that there was sometimes too much feedback from different sides, which led to ambiguity, delays and doubts. "I found it difficult to deal with the sometimes contradictory recommendations" (quote student). That is why, since the third MTA, we have adjusted the role of the student's own supervisor. In case of uncertainty and doubt, the first supervisor, together with the student, has to make choices and substantiate them. "It helps to have regular individual consultations with your supervisor during the first semester in order to make some decisions" (quote student). After all, within Ghent University the evaluation criteria of the student's own educational programme continue to apply.

Students experienced the workload within an MTA differently. Some indicated that the many collective moments made for a too time-consuming process that was not always in proportion to the benefits, especially in the difficult COVID-19 times with energy-consuming online sessions. “When the group was big and the workshop only lasted 1.5 h, I sometimes missed the depth of the discussion of my own master's thesis" (quote student). Others indicated that the balance is quite perfect. "It is an intensive trajectory, but the benefit was very big for me" (quote student). One student also indicated that there is still room for improvement within an MTA "if certain data collection about the case is done jointly by all students".

It remains of great importance that the MTA functions as a socially safe space where there is openness and respect for each other's perspectives and logics, as well as a readiness for dialogue among equals. Students are positive about the lack of hierarchy within an MTA. "The setting feels informal. Everyone is addressed by their first name" (quote student). In our MTAs, we observed that many actors, certainly students, make themselves vulnerable by sharing sensitive data and sometimes using conflicting discourses. The fact that, as a broad community, we still have to take some steps in this area was also demonstrated this year when a few committed MTA students accompanied a group of activists in the neighbourhood Sint-Bernadette and subsequently saw their request for an interview refused by an urban incumbent. Although the latter is sympathetic to the *Stadsacademie* and the MTA, he found it difficult to start a conversation with these students for fear that confidential information would leak out to the activists and squatters in this neighbourhood. Fortunately, we could clear the air between the actors involved and translate this dispute into an interesting learning moment. We have made it explicit that students cannot take a stand on behalf of the MTA, but they can, of course, do so in their own name. The MTA must remain a place that carries out an inter- and transdisciplinary analysis of specific WREST problems relating to social housing, but also a safe place that allows us to learn to develop a well-founded position, even if this does not correspond to the vision of incumbents.

As indicated in Sect. 2.2, a WREST problem, and therefore an MTA, lends itself perfectly to confronting students with a plurality of perspectives, framings and interests, but this must not lead to an ‘anything-goes’ relativism or acquiescence. We therefore explicitly ask the students to develop a substantiated position on the issues at stake and to engage into a constructive, politicised debate about it. Sometimes this leads to a tense relationship with other stakeholders (see above), sometimes it succeeds in (re)politicising certain issues (see Sect. 5.3). However, most students remained cautious in their 'political engagement', a few took more radical steps and assumed an active role. "I have experienced how social problems are structured […] The housing crisis forced me to become an activist, as part of the collective. […] Later, when I started writing, I again and organically took on more of a role as a participatory observer" (quote student).

### Re-integration of the insights in both scientific and societal practices (Phase 3)

Within the MTAs, this phase currently remains the greatest challenge. The nature of the most visible end products, namely master theses, do not appear to be the ideal format to reintegrate the knowledge to the urban stakeholders back or even spread it among universities. As indicated in Sect. 4.1, master theses should follow the standards, design and timing of the specific educational programs. Although the quality control of the MTA master theses in some programs (i.e. Engineering-Architecture and Urbanism and Spatial Planning) is already extended by involving certain external urban actors in the assessment and defence of the master thesis, the focus still remains too much on monodisciplinarity and scientific credibility, and thus not on transdisciplinarity and direct relevance to the stakeholders involved in the atelier. We do post the final results in an attractive archive on the *Stadsacademie* website, but a strong valorisation process also needs other forms of societal outreach.

Urban stakeholders do indicate that "a lot of time is invested in MTAs and both the city and the local citizens sometimes expect a bit more feedback on the final results" (quote urban stakeholder). Although in recent years several feedback moments were cancelled because of COVID-19, this concern remains an agenda item that is closely followed up in the steering committee in which academic and non-academic actors participate (see Sect. 4.1).

In any case, we were able to organise an event in October 2021 at which the main results of the past academic year were presented by the graduates to all urban stakeholders involved and to the new batch of MTA students. So at this event, we are handing over the so-called relay baton and hope to continue the journey of the MTA. Every two or three years this will also be accompanied by an exhibition about the entire MTA ‘Diversity in Social Housing’ in cooperation with the city of Ghent.

We realise that for now the impact is less visible in the end products, but more present in the broader approach of the *Stadsacademie* program. At the academic level, MTAs have already led to some joint project proposals. Within a MTA, a network is formed, an issue is explored, and then a consortium develops a research proposal. At the policy level, we manage to get certain themes on the political agenda through a MTA. For example, the MTA in 2019–2020 ensured that there was a thorough reflection on the nature of the social mix in the neighbourhood ‘Nieuw Gent’. “The *Stadsacademie* has ensured that a fixed idea is brought back into discussion” (quote urban stakeholder). And as already mentioned, in the ongoing MTA, a spirited discussion arose between policy makers on the one hand and activists and MTA students on the other, in which the MTA coordinators were ultimately able to play an interesting mediating role. So not only is the voice of the *Stadsacademie* being heard more and more, but the setting is also proving to be that free-thinking space where conflicting discourses can have a constructive dialogue.

The above already makes it clear that the MTAs are fragile environments that require diplomatic key figures who continue to keep an eye on the original philosophy of the *Stadsacademie*. Neither should the students and their supervisors lapse into a consultant role for the city, nor should they completely evade the valorisation expectations of the urban stakeholders. At the same time, all the actors involved must be able to make themselves vulnerable (for example, by taking clear normative positions) without being called to account for this later on.

## Overall reflections and conclusions

While in the previous section we mainly referred back to the three-phase model of Lang et al. (2012), we would like to structure the concluding section largely on the main characteristics of Mode 2 Science (cf. Tables 1 and 2). Given the central aim of this article, we reflect on 10 general lessons or suggestions on how transdisciplinary settings with master theses can address complex urban sustainability issues.

1. **Tackle a specific urban WREST problem**

Students work best on a specific and wicked ‘real world problem’ to experience how and why each time a plurality of perspectives struggle (O'Leary, 2005; Östman et al., 2019; Walker et al., 2015). In this way, the lack of a 'correct' problem definition and clear-cut solutions makes it clear that knowledge is often incomplete and uncertain, that different interpretations are possible, that there may be discussion among scientists, that imaginaries of the future may differ, etc. Ideally, a controversy surrounding a WREST problem should not be understood as problematic, but rather the opportunity should be seen to open up space for an interesting debate (Block et al., 2019; see also number 2). Diving into a real world problem also often confronts students with issues they usually have no experience with: conflict, poverty, outrage, etc. For this reason, among others, there is also a need for a strong ethics of care within the *Stadsacademie* (see also number 5). Urban actors indicated that a MTA on a WREST problem would allow them to explore issues of which they do not even know the relevant questions yet, let alone launch a typical call for consultancy around this issue already.

2. **Take serendipity as one of the guiding principles**

Trajectories within the *Stadsacademie* therefore mainly have an exploratory character in which we keep the questions open and do not fix the outcomes. MTAs allow us to learn together in an open and experimental way about a WREST problem. Hence, the concept of ‘collaboratorium’. In this sense, students’ work may not be tied to an obligation to follow a strict format or produce a predefined result. The serendipity lies in the hope that, without a specific and fixed search, we will find interesting and possibly unexpected angles and solutions. However, this also means that a win–win situation for all parties involved (see also number 9) is not a-priori guaranteed. Failure is an option and we see this—if there is room for continuous reflection—also as a learning opportunity.

3. **Recognise the importance of multiperspectivism and context-specificity**

Addressing a specific WREST problem in an open and transdisciplinary collaboratorium should allow for different perspectives. As such, students can learn how to deal with a plurality of scientific positions, ontologies, framings, normative perspectives and ethical values (Evans, 2015; Östman et al., 2019; Wiek et al., 2011). It is fascinating to see that in this way most students also immediately recognise their own blind spots and understand that their acquired knowledge is context-specific: it is linked to a specific non-neutral theoretical and analytical lens as well as to a specific spatial and temporal scale (Evans, 2015; Jasanoff, 2004; Nowotny et al., 2005). This is also one of the great (policy) challenges we face: learning to navigate agilely between these different scales in the search for context-specific answers to complex sustainability issues. What makes sense when?

4. **Dare to engage politically**

The awareness of how knowledge is produced and the presence of different perspectives should not lead to ‘anything-goes’ relativism or resignation, but rather should open up the possibility of taking an engaged position. Precisely because knowledge production around a WREST problem is ‘political’ (Block et al., 2018; Jasanoff, 2004), it is important that students (and all other actors involved in the MTA) also dare to engage politically, especially since it often involves an urgent challenge of urban sustainability. That is why we invite students to relate to the WREST problem, take responsibility for it, and adopt a substantiated position. However, it is important to keep the previous suggestion (number 3) in mind.

5. **Make sure the transdisciplinary setting is a ‘safe space’**

A shift from 'neutral observers' (Mode 1) to 'reflective and engaged researchers' (Mode 2) makes it clear that both students and all other actors involved make themselves vulnerable by admitting that their perspectives and substantiated position are not universally correct, but arise from a specific context, concern, scale and theoretical-analytical perspective. In particular, taking a normative position around a real problem requires some courage. In this sense, it is important that all actors involved experience trust and safety to dare to step out of their respective comfort zones.

It remains a permanent challenge for the coordinators of the MTA to guarantee this safety and a strong ethics of care, to ensure respect for each other's perspectives and points of view and to maintain the existing networks and relationships based on trust. Fortunately, it is becoming increasingly clear that the *Stadsacademie* can effectively function as that unique safe place and, even more so, as that free space where a dialogue can take place in complete confidence between parties who have long been at odds.

6. **Expect time-intensive processes, but embrace slowness**

Building such a safe space takes time. This is true for the *Stadsacademie* as a whole, but equally true for a MTA as a crucial part of a trajectory. Time and again, a great amount of energy must be put into developing the philosophy and approach as well as transdisciplinary processes and networks in which trust is present to speak openly and in confidence. We therefore suspect that it is less obvious to initiate a similar initiative such as the *Stadsacademie* top-down and quickly. A MTA seems to benefit from organic growth, the connection of processes step by step, the linking of existing networks, the learning by doing in terms of general philosophy and focus, the time we take to get to know each other's assumptions and interests, etc. Hence our interest in the idea of ‘slow science’ (Stengers & Muecke, 2017). Within the more tangible aspects of a MTA, we experienced the time-intensive nature in the three phases of the transdisciplinary process (cf. Sect. 5), especially within the formation of a supervisor network from a fragmented university characterised by different procedures, regulations and expectations around master's theses (Balsiger, 2015; Evans, 2015). The logistical preparation of site visits, speed dates, workshops and debates should also not be underestimated. The choice to run a MTA over several years can also be understood as a choice for slowness. We don’t try to capture the WREST problem all at once, but in line with the idea of serendipity to walk a journey where students build on each other's work year after year.

7. **Be alert for dominant Mode 1 logics**

We certainly do not want to minimise the importance of Mode 1 Science, but the dominance of this way of knowledge production does not always make it easy for the *Stadsacademie* and MTAs. The Mode 2 Science of the Stadsacademie requires a lot of time and energy, some modesty and engagement, as well as the acknowledging uncertainty and serendipity, multiperspectivism and context-specificity (see number 2–4). When the comfort zone is left, it is sometimes tempting to fall back on the more common and system-conforming Mode 1 Science. And of course, the compartmentalised structures within universities don't help MTAs develop easily either.

We also understand the scepticism of authors who point out mechanisms of inclusion and exclusion within inter- and transdisciplinary settings (Hessels & van Lente, 2008; Viseu, 2015). Whose and which knowledge counts in the end? Because co-production processes are inherently political and contested arenas in which different interests, norms, perspectives and (unequal) power relations converge (Felt, 2007; Turnhout et al., 2020), we need to be careful that non-academic knowledge (or knowledge from the social sciences) is not limited to lip service within what then ultimately remains, for example, a dominant technoscientific 'Mode 1' narrative.

8. **Continue to learn about transdisciplinarity**

We cannot expect MTA participants (including coordinators) to learn about transdisciplinarity just by being present in the process. Our MTAs show that in addition to thematic workshops and meetings, there is also a need for workshops where the transdisciplinary process itself is discussed and evaluated with all stakeholders. On a general level within the *Stadsacademie*, a working group has been established to continue learning about transdisciplinarity by organising lectures, debates and workshops around cognitive aspects (e.g. dealing with different ontologies and epistemologies), methodological aspects (e.g. interesting transdisciplinary frameworks or tools) and/or institutional aspects (e.g. coordination, broad communication and archiving).

9. **Be clear about roles, responsibilities and expectations**

All academic and non-academic actors participate voluntarily in the MTA and in all cases this is an additional and not to be underestimated commitment on top of full agendas. It is therefore important to create a win–win situation for everyone. Since the logics and interests of students, supervisors, policy makers and other non-academics differ, this is not self-evident. Because the students in the *Stadsacademie* receive a unique setting and guidance, they are certainly a direct beneficiary, but we must continue to ask ourselves whether a MTA is too demanding for them and there is sufficient return. The same applies to supervisors and other academics: they may be able to experiment in transdisciplinary settings and expand their networks, but they are also expected to participate in all MTA initiatives and also provide constructive feedback on the work of all students (not just the student work they promote). And, of course, the City of Ghent is also intended to benefit from a MTA. The latter does not fit into a consultancy logic, but a return for the investments of all urban actors does remain a goal. It is therefore essential to communicate well from the outset about the overall set-up, roles, responsibilities and expectations (cf. Section 5.2). Those who step on board should know what to expect. Then, when everyone agrees and a good division of tasks is in place, a broad and shared ownership can grow.

10. **Do not underestimate the importance of coordinators**

We have already mentioned the importance of organic growth and serendipity, but a strong organisation is equally important. A good balance between the organic and the organisational is key. In addition to a pleasant and open location for the MTA, committed actors and networks, and an interesting philosophy and approach, it is important to have an appropriate organisational structure that allows sufficient room for flexibility. Our experience shows that there is a need for 1 or 2 coordinators per MTA, but also for a general coordinator who acts as a general point of contact, organises the selection of new WREST problems, ensures coordination between the MTAs, stimulates learning about transdisciplinarity, takes care of general communication and the website, etc.

We hope that both the above 10 lessons and the specific description of the MTAs ‘Diversity in Social Housing’ can inspire transdisciplinary approaches within educational practices to address complex urban sustainability issues. In doing so, we should not be discouraged by the fact that these time-intensive and challenging trajectories rarely lead to clear-cut answers to WREST problems. Transdisciplinary processes are rather never-ending stories (Rittel & Webber, 1973; Max-Neef, 2005) characterised by a temporary grasping, a respectful interweaving of each other's perspectives, and several political engagements in pursuit of a more sustainable city.

## Data Availability

The datasets generated during this study are not publicly available due to privacy restrictions, but the transcripts of the focus groups are available from the corresponding author on reasonable request.

## Footnotes

1 In order to emphasise the particularity and ownership of our Ghent initiative as much as possible, we opt to use the Dutch-language name, also in English publications. A good translation is ‘Urban Academy’, but this concept is used worldwide for similar and different initiatives.

2 See also www.destadsacademie.be for an accurate overview of the trajectories.
